# Novel Biodegradable Nanoparticulate Chain-End Functionalized Polyhydroxybutyrate–Caffeic Acid with Multifunctionalities for Active Food Coatings

**DOI:** 10.1021/acssuschemeng.3c00389

**Published:** 2023-04-27

**Authors:** Fady Abdelmalek, Marian Rofeal, Joanna Pietrasik, Alexander Steinbüchel

**Affiliations:** † International Center for Research on Innovative Biobased Materials (ICRI-BioM)—International Research Agenda, Lodz University of Technology, Zeromskiego 116, Lodz 90-924, Poland; ‡ Department of Botany and Microbiology, Faculty of Science, Alexandria University, Alexandria 21521, Egypt; § Faculty of Chemistry, Institute of Polymer and Dye Technology, Lodz University of Technology, Stefanowskiego 16, Lodz 90-537, Poland

**Keywords:** functional PHB, caffeic acid, green nanoparticles, polymer modification, food coating

## Abstract

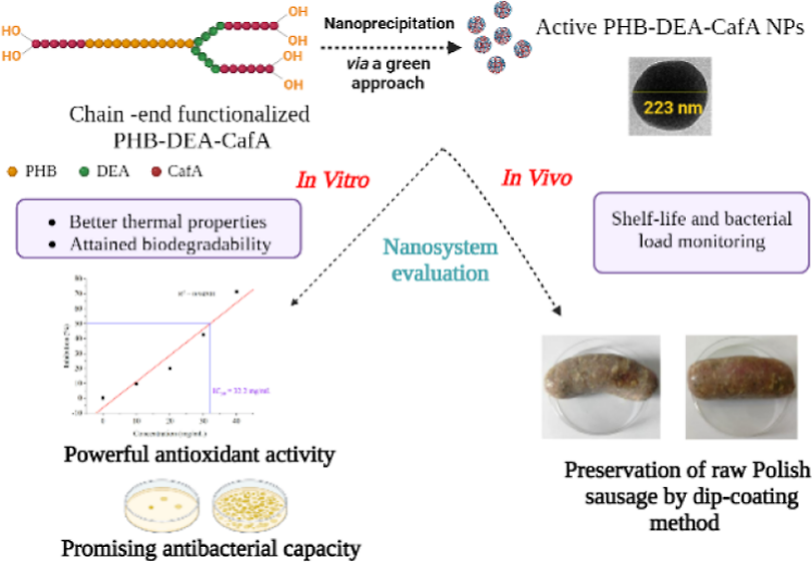

The bioactivities of polyhydroxyalkanoates have been curtailed owing to the lack of bioactive functional groups in their backbones. In this regard, polyhydroxybutyrate (PHB) produced from new locally isolated *Bacillus nealsonii* ICRI16 was chemically modified for enhancing its functionality, stability as well as solubility. First, PHB was transformed to PHB-diethanolamine (PHB-DEA) by transamination. Subsequently, for the first time, the chain ends of the polymer were substituted by caffeic acid molecules (CafA), generating novel PHB-DEA-CafA. The chemical structure of such a polymer was confirmed by Fourier-transform infrared (FTIR) spectroscopy and proton nuclear magnetic resonance (^1^H NMR). The modified polyester demonstrated improved thermal behavior compared to PHB-DEA as was shown by thermogravimetric analysis, derivative thermogravimetry, and differential scanning calorimetry analyses. Interestingly, 65% of PHB-DEA-CafA was biodegraded in a clay soil environment after 60 days at 25 °C, while 50% of PHB was degraded within the same period. On another avenue, PHB-DEA-CafA nanoparticles (NPs) were successfully prepared with an impressive mean particle size of 223 ± 0.12 nm and high colloidal stability. The nanoparticulate polyester had powerful antioxidant capacity with an IC_50_ of 32.2 mg/mL, which was the result of CafA loading in the polymer chain. More importantly, the NPs had a considerable effect on the bacterial behavior of four food pathogens, inhibiting 98 ± 0.12% of *Listeria monocytogenes* DSM 19094 after 48 h of exposure. Finally, the raw polish sausage coated with NPs had a significantly lower bacterial count of 2.11 ± 0.21 log cfu/g in comparison to other groups. When all these positive features are recognized, the polyester described herein could be considered as a good candidate for commercial active food coatings.

## Abstract 2

The development of this novel biodegradable nanoparticulate chain-end functionalized polyhydroxybutyrate−caffeic acid with multifunctionalities for active food coatings is an important step toward sustainable food packaging, as it provides a biodegradable and multifunctional material that can help reduce food waste and its environmental impact.

## Introduction

Despite the numerous merits of polyhydroxyalkanoates (PHAs) due to their biocompatibility, biodegradability, and nontoxicity, the lack of their chemical functionalities and poor mechanical properties have stifled their wide implementation in the industrial and biological sectors.^1^ Hence, there has been a pressing need for novel sustainable polymer variance with ameliorated capacities.^2^ In this respect, several investigations have studied the possibility of PHA surface modification through binding to tumor or bacterial specific ligands to widen their applications in the medical and food sectors.^3^ In fact, the chemical polyester modification methods try to alter their backbones and their characteristics while maintaining their biodegradation. PHAs’ functionalization has proven to be a more effective approach to enhance their bioactivities and mechanical properties by adding active chemical constituents to the polymeric structures.^4^ For instance, Haraźna et al. has recently functionalized PHA with tricalcium phosphate through modification of diclofenac oligomers, generating a new functional bone tissue substitute.^5^

Phenolic acids are deemed as promising candidates for inactive polymer functionalization because of their antioxidant, antimicrobial, and anticancer properties as well as safety. Among these, caffeic acid (CafA) (3,4-dihydroxy cinnamic acid) is a highly abundant phenolic molecule found in many plant products, including fruits, legumes, and wine.^6^ Due to CafA’s strong antioxidant activity,^7^ it has been proven that consumption of CafA-containing plant products improves health and protects against disease.^8^ CafA has been widely used to treat cancer and other infectious disorders caused by viral, bacterial, and fungal pathogens.^9,10^ CafA has been also studied for food preservation against *Staphylococcus aureus*, with the antibacterial activity being attributed to the presence of multiple hydroxyl groups on its benzene ring.^11^ To solve the issue of its poor water solubility, it has been encapsulated or grafted onto polymeric molecules with the aim of diversifying polymer structures.^6^ On another avenue, transforming the activated polymers to the nanoform would be an extraordinary way for not only enhancing the phenolic acid solubility and bioavailability but also improving the polymer processability for food safety applications.^12^

Active packaging research has grown recently because of its capacity to prevent degradation and enhance quality of food. The combination of product safety, increased shelf life, and antioxidant capabilities has heightened interest in active packaging integrated with bioactive compounds.^13,14^ Food coatings have the ability to prevent or reduce the migration of moisture, oxygen, and carbon dioxide. They have the capability of transporting food additives including flavorings, antioxidants, antimicrobials, as well as improving food handling properties and mechanical integrity.^15^ Meat and meat products, such as sausages, are prone to microbial contamination and oxidative deterioration, which could be detrimental to general health.^16^ Food safety can potentially be impacted by microorganisms.^17^ Fortunately, effective food packaging, such as coatings and edible films, can overcome such issues.

The cost of PHA production has limited its application in the functional food sector.^18^ The genus *Bacillus* appears to be a promising choice for polyhydroxybutyrate (PHB) synthesis owing to higher polymer productivity and less demanding fermentative parameters.^19^ The *Bacillus* genus’ unique PHA synthase implies that the genus could be a prospective producer of both novel and recognized PHA with varied monomeric contents.^20,21^

In this study, a three-arm hydroxylated PHB-diethanolamine (PHB-DEA) was synthesized and functionalized for the first time with CafA. The generated novel polyester was examined for its thermal behavior and biodegradation profile compared to native PHB. Moreover, green polymeric vehicles have been developed and tested for their antioxidant and anti-food pathogen efficacies. Then, the nanocarriers have been employed as food coating in vivo using raw meat/pork sausage samples. To the best of our knowledge, to date, this is the first investigation proposing chain-end functionalized PHB with CafA for food preservation potentials.

## Materials and Methods

### Materials and Chemicals

Nile blue stain, diethanolamine (DEA) (98%), 4-dimethylaminopyridine (DMAP) (99%), *N*,*N*-dicyclohexylcarbodiimide (DCC) [99% (GC)], stannous 2-ethyl hexanoate (Sn-oct) (92.5–100%), dimethyl formamide (DMF) (99%), dichloromethane (DCM) (95%), caffeic acid (CafA) [99.0% (HPLC), MW: 180.16 g mol^–1^], and 2,2-diphenyl-1-picrylhydrazyl (DPPH) were provided from Sigma-Aldrich, Germany. Peptone water, Tryptic Soy Agar (TSA), and Tryptic Soy Broth (TSB) were obtained from Pol-Aura, Poland. The other chemicals and solvents were purchased from Alchem, Poland. DSMZ-German Collection of Microorganisms and Cell Cultures GmbH, Germany, provided the bacterial strains utilized to test antibacterial activity including *S. aureus* DSM 683, *Listeria monocytogenes* DSM 19094, *Salmonella enterica* DSM 9386, and *Escherichia coli* DSM 787. Fresh sausages, Biała kiełbasa (Pork mush 91.8%, beef meat 2.5%, salt, and spices 0.3%) were obtained from retail stores in Lodz, Poland.

### Isolation of Polyester Bacterial Producers

Soil samples were collected from different locations on the campus of Lodz University of Technology, Lodz, Poland. Sterile containers (100 g) were used to collect samples, which were then brought immediately to the lab and serially diluted before the PHA screening plates were inoculated. Linko medium with 0.025 mg/L Nile blue stain (Sigma-Aldrich, Germany) was used for PHA screening. Colonies of orange color were selected and preserved after exposure to UV light.^22^ The screening Linko medium is composed of (g/L); glucose (15), KH_2_PO_4_ (2), (NH_4_)_2_SO_4_ (2), yeast extract (0.75), MgSO_4_ (0.75), and agar (15). For future use, single colonies of PHA-producing bacteria were grown for 24 h on nutrient agar plates (pH 7.0 at 37 °C). With a few minor changes, Abdelmalek et al.^19^ previously described the medium used for the synthesis of PHA. The production medium included glucose (20 g/L), (NH_4_)_2_SO_4_ (0.5 g/L), MgSO_4_ (0.8 g/L), Na_2_HCO_3_ (0.5 g/L), Na_2_HPO_4_ (2.0 g/L), KH_2_PO_4_ (2.0 g/L), CaCl_2_ (0.05 g/L), and 5 mL trace element solution containing MnCl_2_·4H_2_O (0.003 g/L), CoCl_2_·7H_2_O (0.02 g/L), H_3_BO_4_ (0.003 g/L), NICl_2_·6H_2_O (0.002 g/L), ZnSO_4_·7H_2_O (0.01 g/L), and CuCl_2_·2H_2_O (0.001 g/L). The fermentation process was conducted at 180 rpm, pH 7 at 37 °C for 96 h. The extraction and purification of the polyester produced was carried out according to our previous report.^23^ Fourier-transform infrared (FTIR) spectroscopy analysis was performed to detect the characteristic functional groups of the generated polyester.

### Molecular Identification of the Selected PHA Producer

The extraction of the bacterial genomic DNA from the selected isolate was performed using Genomic Mini kit (A&A biotechnology, Poland). The cell pellets were incubated with lysozyme at 37 °C for 20 min. By using an MJ Mini Gradient Thermal Cycler, polymerase chain reaction (PCR) was performed (Bio-Rad, Hercules, CA, USA). The universal primers 27F and 1492R (5′-AGAGTTTGATCCTGGCTCAG-3′/5′-GGTTACCTTGTTACGACTT-3′) were employed to amplify the 16S rRNA gene. The PCR reaction was carried out according to our previous report.^19^ The 16S rRNA gene’s nucleotide sequences were edited, put together, and aligned. Moreover, the MEGA 11 sequence alignment software (version 11.0.11) was used to generate consensus sequences, which were then tested using the BLAST program. The 16S rRNA gene’s nucleotide sequences were utilized to establish genetic connections using the neighbor-joining method.^19^

### Preparation of PHB-DEA

**Scheme 1:**
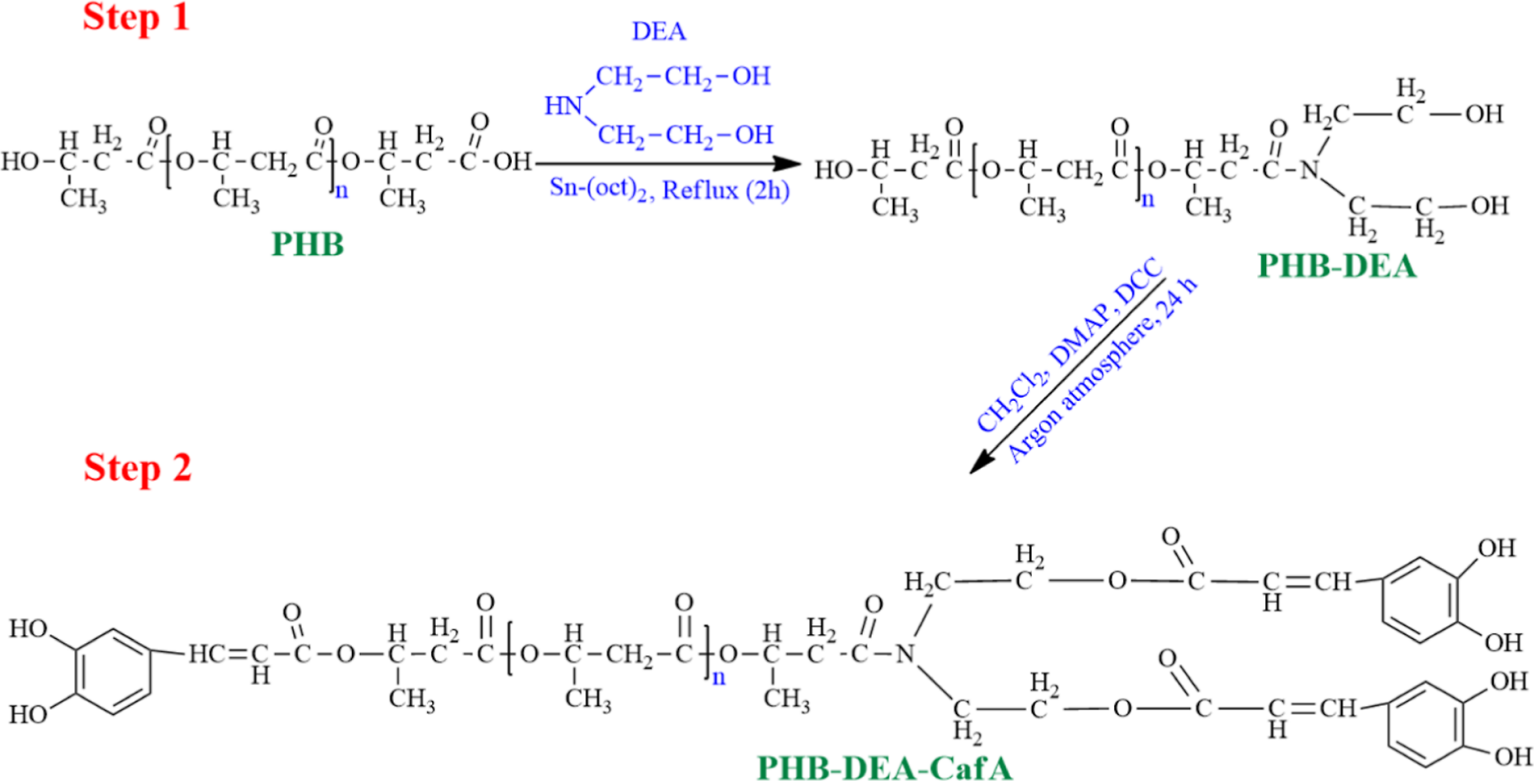
Two-Step Chain-End Functionalization of PHB with CafA

The transamination reaction of PHB with diethanolamine (DEA) is displayed in the first step of Scheme 1. PHB was transaminated with DEA in chloroform under reflux conditions. In order to demonstrate the transamination process, 100 mL of chloroform was mixed with 4 g PHB and 0.03 g tin (2-ethyl hexanoate), then the mixture was refluxed for additional 2 h. Subsequently, 4 g of DEA was introduced into the mixture, which continued to reflux for another 2 h. The polymer was obtained after solvent removal by rotary evaporator and washed using a petroleum ether before being vacuum-dried overnight at 40 °C. The white polymer powder obtained was cured for 1 h at 110 °C to finish the transamination process. After being redissolved in 50 mL of chloroform, the dried polymer was subsequently recovered in 300 mL of methanol. The final product of PHB-DEA underwent filtration and vacuum drying for 12 h at 45 °C.^24^

### Chain-End Functionalization of PHB-DEA by CafA

Using the esterification process described by Hazer et al.,^25^ PHB-DEA and CafA reacted to generate PHB-DEA-CafA. PHB-DEA (3.5 g) was dissolved in dichloromethane (DCM) (30 mL). CafA (2.5 g), dicyclohexylcarbodiimide (DCC) (3.0 g), and 4-dimethylaminopyridine (DMAP) (0.3 g) were added to the solution under continuous stirring and argon atmosphere for 24 h. Then, dihexyl urea was precipitated as a side product. The precipitate was filtered, the solvent of the filtered solution was evaporated, and the crude product was leached with excess of methanol, where the PHB-DEA-CafA was purified via filtration. The white solid product was dried under vacuum at 40 °C for 24 h.

### FTIR

The chemical structures of CafA, PHB-DEA and PHB-DEA-CafA were analyzed in 400–4000 cm^–1^ range using FTIR Nicolet 6700 spectrophotometer (Thermo Scientific, USA).^26^

### Nuclear Magnetic Resonance (^1^H NMR)

The ^1^H NMR analyses for the obtained products were performed using Agilent NMR 600 MHz NMR (Agilent, Santa Clara, CA, USA) spectrometer at 25 °C. Samples (5 mg) of PHB-DEA and PHB-DEA-CafA were dissolved in 1 mL deuterated chloroform (CDCl_3_). The acquisition parameters were as follows: 14 ppm sweep width, 0.1 s pulse delay, continuous WALTZ-16 broadband 1H decoupling, 1.7 s acquisition time, and 45° hard pulse angle. Each sample received up to 2000 scans, which equated to 1 h of collection time.^27^

### Molecular Analysis

Gel permeation chromatography (GPC) was used to determine the molecular weights of PHB, PHB-DEA, and PHB-DEA-CafA using a Wyatt instrument (Wyatt, Dernbach, Germany) fully equipped with light scattering (LS), one guard column [GRAM Linear (10 m, *M*_n_ between 800 and 1,000,000 Da)], differential refractometer (RI) (Wyatt, Dernbach, Germany), and two Perfect Separation Solution (PSS) columns. At a flow rate of 1 mL/min, 1 mg of sample was dissolved in 1 mL of DMF with 50 mmol LiBr as eluent. Calibration was performed using poly(methyl methacrylate) or polystyrene (PS) as standards.^28^

### Thermal Properties

The thermal stabilities of the native and synthesized polymers were investigated by thermogravimetric analysis (TGA) using Mettler Toledo TGA Star* system (Ohio, USA). Temperatures ranged from 30 to 600 °C, with a heating rate of 10 °C/min in a nitrogen environment (flow rate of N_2_ = 20 mL/min) were the conditions for the analyses. The samples’ degradation rate was detected by analyzing temperature data from TGA and derivative thermogravimetry (DTG) analyses at *T* 5, 10, and 50%.^22^

Differential scanning calorimetry (DSC) analysis using Mettler Toledo1 series (Ohio, USA) was used to measure the melting point (*T*_m_) and glass transition temperatures (*T*_g_) of polymer samples in N_2_ environment with flow rate of 20 mL/min. For DSC analysis, 3.0 mg of PHB, PHB-DEA, and PHB-DEA-CafA were put into an aluminum pan and heated at a rate of 10 °C/min from −10 to 200 °C. The point of inflexion in the DSC curve between the onset and offset temperatures indicates to the glass transition temperature and the melting point, which was reported as the peak temperature of an endothermic event.^29^

### Biodegradability

Several films of PHB, PHB-DEA, and PHB-DEA-CafA were allowed to dry completely for a week before being exposed to clay soil. Film strips were adjusted to 10 × 60 mm and a constant initial weight of 100 mg. Biodegradation tests were performed by burring all film strips in the soil at a depth of 10 cm. After 10, 25, 40, and 60 days, the investigated strips were collected from the soils, rinsed in sterile distilled water, and dried at 25 °C for one day, and then weighed.^30^ The degradation of the polymeric strips was typically assessed and compared in terms of percentage weight loss using the following equation:
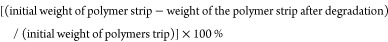


### Preparation and Physicochemical Characterization of PHB-DEA-CafA NPs

The polymer powder was dissolved in glacial acetic acid at 110 °C for 20 min to create a stock solution containing 0.5 wt % PHB-DEA-CafA. To get rid of any undissolved polymer, the produced solution was filtered using a Teflon 0.45 m filter. The PHB-DEA-CafA NPs were generated via nanoprecipitation by continuously stirring at 1500 rpm, while injecting 30 mL of the filtered stock solution into 15 mL of the receiving solution (2 g NaOH in 100 mL DW).^31^ For determining the zeta potentials and the particles size distribution, the Malvern Zetasizer (NanoZlS/ZEN3600 Zetasizer Malvern Instruments Ltd., UK) was used. The diffractive index was set at 2.5 and the temperature was kept at 25 °C. Transmission electron microscopy (TEM) (JEM-2100F-JEOL, 200 kV, Japan) was used to investigate the morphology of PHB-DEA-CafA NPs. Before analysis, the NPs solution was diluted with deionized water (1:100 v/v), and a sample drop was placed on a copper grid, stained with uranyl acetate solution for 30 s, and gently dried.^32^ The CafA content in PHB-DEA-CafA NPs was determined by UV–vis spectroscopy at 325 nm.

### Bioactivities of PHB-DEA-CafA NPs

**Figure 1:**
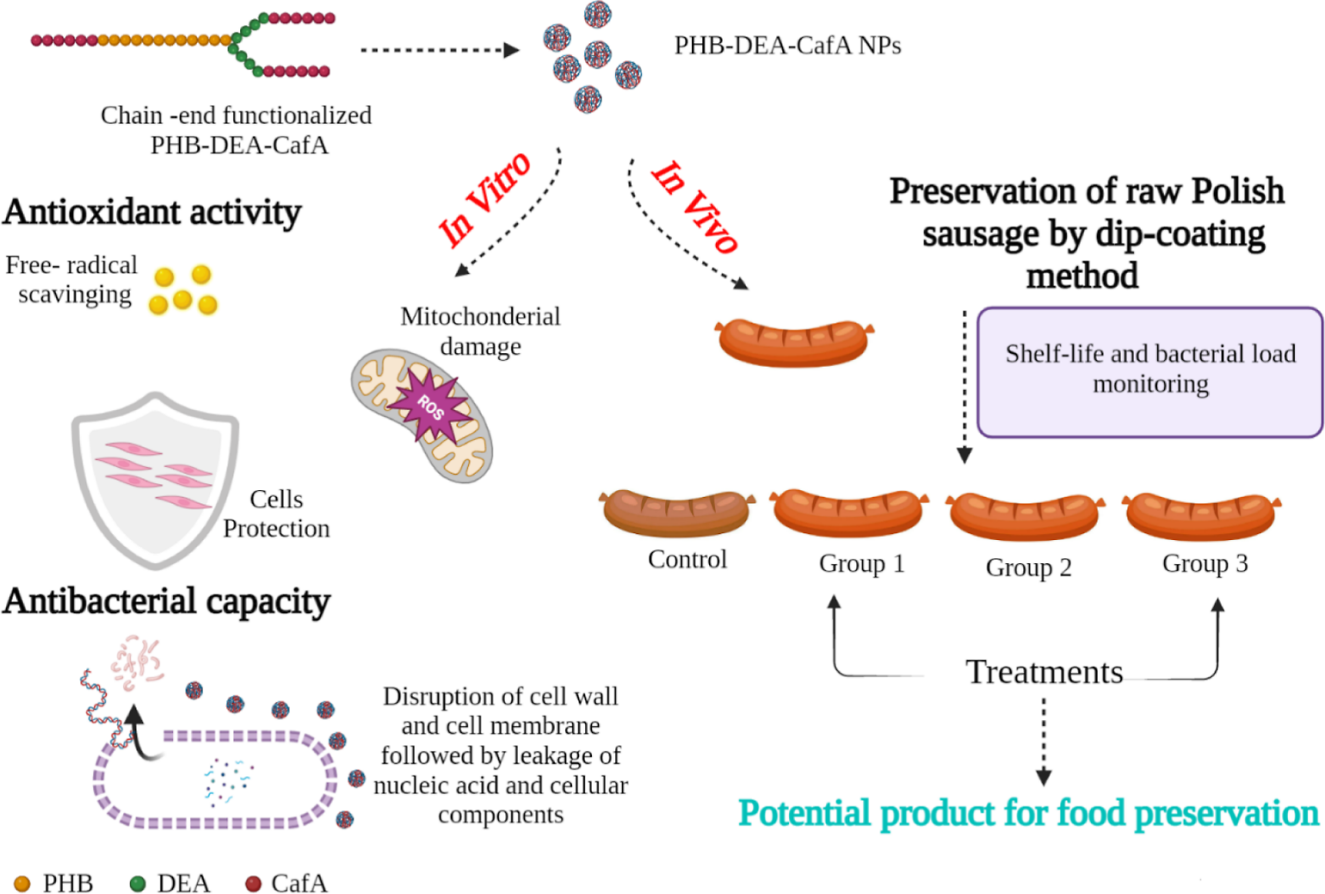
Schematic illustration of the multifunctionalities of the newly synthesized PHB-DEA-CafA NPs.

The bioactivities of the newly functionalized polyester (PHB-DEA-CafA NPs) were investigated in terms of free radical scavenging capability and antibacterial capacity for food safety potentials (Figure 1).

### In Vitro Appraisal of PHB-DEA-CafA NPs

#### Antioxidant Activity Using DPPH Free Radical Scavenging Activity

The antioxidant activity of PHB-DEA-CafA NPs was determined following the protocol of Pérez-Jiménez and Saura-Calixto^33^ with some modifications. Briefly, different concentrations of PHB-DEA-CafA NPs were dissolved in 1 mL of methanol, stirred for 3 h at 150 rpm, and then centrifuged for 10 min at 4500 rpm. For the DPPH radical assay, 4 mL of a 0.004% DPPH in methanol solution was combined with 1 mL of each polymeric NP at different concentrations (10–40 mg/mL). The samples were then incubated in the dark at 25 °C for 30 min. The scavenging capability was measured using a spectrophotometer (Thermoscientific MultiskanTM, Singapore) at 517 nm. Ascorbic acid was utilized as a standard, and a control mixture was made without NPs. The DPPH inhibition percentage (*I* %) was then calculated as follows:
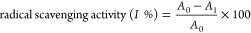
*A*_1_ is the absorbance of the NP samples, while *A*_0_ is the absorbance of the control.

The NPs concentration that provided 50% of the radical scavenging activity (IC_50_) was estimated using the graph of radical scavenging activity percent versus NPs concentration.^34^

#### Antibacterial Capacity

The antibacterial activity of PHB-DEA-CafA NPs with different concentrations (1, 2.5, and 5 mg/mL) was detected using the plate counting method against four food pathogens, which were *S. aureus* DSM 683, *L. monocytogenes* DSM 19094, *S. enterica* DSM 9386, and *E. coli* DSM 787. Sterile flasks containing 100 mL of TSB medium were inoculated with 5 mL of each tested bacterial suspension (0.5 McFarland 1.5 × 10^8^ CFU/mL), then aliquots of the prepared solutions were added to the flasks. The positive control lacked the treatment solutions and included 5 mL of bacterial suspension in 100 mL of TSB medium. All flasks were incubated for 18–24 h at 37 °C under shaking conditions at 150 rpm. Aliquots from each flask (100 μL) were spread on TSA plates at different time intervals (8, 12, 24, and 48 h), which were then incubated for 18–24 h at 37 °C to determine viable counts. The percentage reduction in the bacterial viability was calculated and expressed in comparison to the positive control group.^26^

### In Vivo Assessment Using Commercial Polish Sausage by Dip-Coating Method

Standard solutions of PHB, PHB-DEA, and free PHB-DEA-CafA were prepared with a concentration of 5 mg/mL in DMSO and stirred for 24 h for complete dissolution, while aqueous solutions with three different concentrations of PHB-DEA-CafA NPs were used (5, 10, and 25 mg/mL). Dip-coating was performed on the commercial sausages by simply dipping the sample thrice at an interval of 5 min in the solution for 15 min. Then, the coated sausages were hung to dry at room temperature. Samples were collected for microbiological analysis every 5 days for the first 30 days of storage at 4 °C. The sausage slices (10 g) were aseptically sliced and homogenized for 3 min in 90 mL of sterile peptone water solution (0.1% w/v) using a stomacher (Stomacher 400, Interscience, France). 100 μL of serial dilutions prepared on agar plates was dispersed on Plate Count Agar to identify total bacterial count (TBC). Total viable counts were determined after 2 days of incubation at 37 °C. The results were interpreted by visually inspecting colonies based on their characteristics (such as form, size, color, etc.) and expressed as log cfu/g of sausage.^35^

### Statistical Analysis

A one-way ANOVA with the Tukey test was used to analyze the statistically significant differences between the investigations (*P* < 0.05 confidence level). The findings of the process monitoring tests were reported as the mean value and its standard deviation. The assays were carried out in triplicate. Prism 7 was utilized to examine the data (GraphPad, Inc.).

## Results and Discussion

### Identification of the PHA Producer

**Figure 2:**
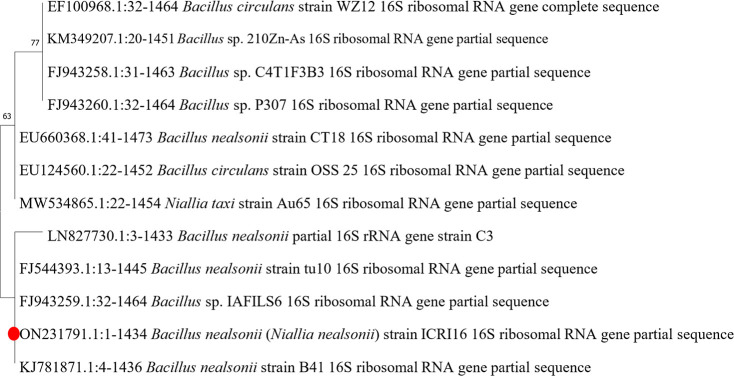
Position of strain *B. nealsonii* ICRI16 in phylogenetic analysis based on 16S rRNA sequences. MEGA 11 sequence alignment editor detected these phylogenetic associations (version 11.0.11).

**Figure 3:**
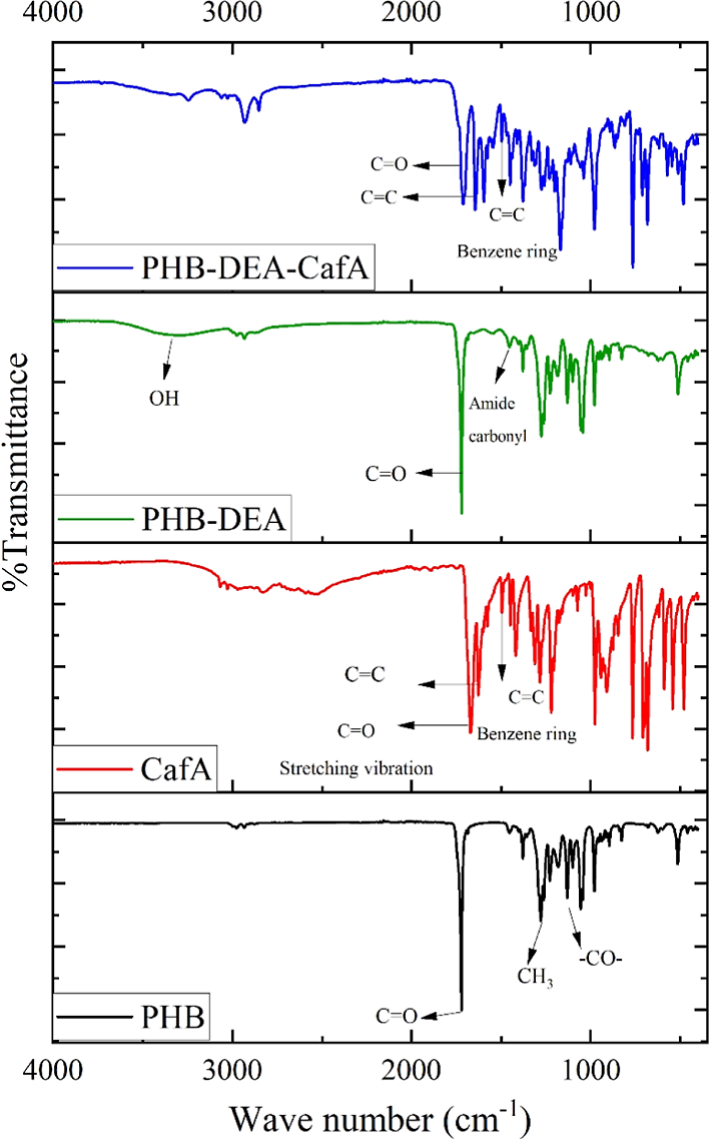
FTIR analysis of PHP produced by *Bacillus nealsonii* ICRI16, CafA, PHB-DEA, and PHB-DEA-CafA.

The PHA producing strain showing orange fluorescence on Nile blue agar plates was selected for additional molecular analysis. The 16S rRNA analysis of the PHA generating strain revealed a high degree of homology to *Bacillus* sp. genera. A sequence analysis using BLAST revealed that strain ICRI16 has a 97% match to *Bacillus nealsonii* (also known as *Niallia nealsonii*). The 16S rRNA gene sequencing of the newly identified strain was submitted to NCBI GenBank as *B. nealsonii* ICRI16. The phylogenetic location of *B. nealsonii* ICRI16 is shown in a neighbor-joining dendrogram with numerous *Bacillus* sp. as an outside group (Figure 2). The 16S rRNA sequence has been submitted in GenBank as accession number ON231791. The FTIR spectra of the extracted polyester revealed a strong absorption peak at around 1119 cm^–1^, which was related to a saturated ester bond of (CO−) groups (Figure 3). Additional absorption peaks at 1350 and 1415 cm^–1^ indicated stretching of the methyl (CH_3_) group. Furthermore, the peaks at 3390, 2915, and 1736 cm^–1^ were the typical peaks of hydroxyl (OH) groups, methine (=CH−), and carbonyl (C=O), respectively. The distinctive group of PHB, which was the common homopolymer of PHAs, was the peak absorbance of the carbonyl group (C=O).^23^

### Characterization of the Chain-End Functionalized PHB-DEA-CafA

**Figure 4:**
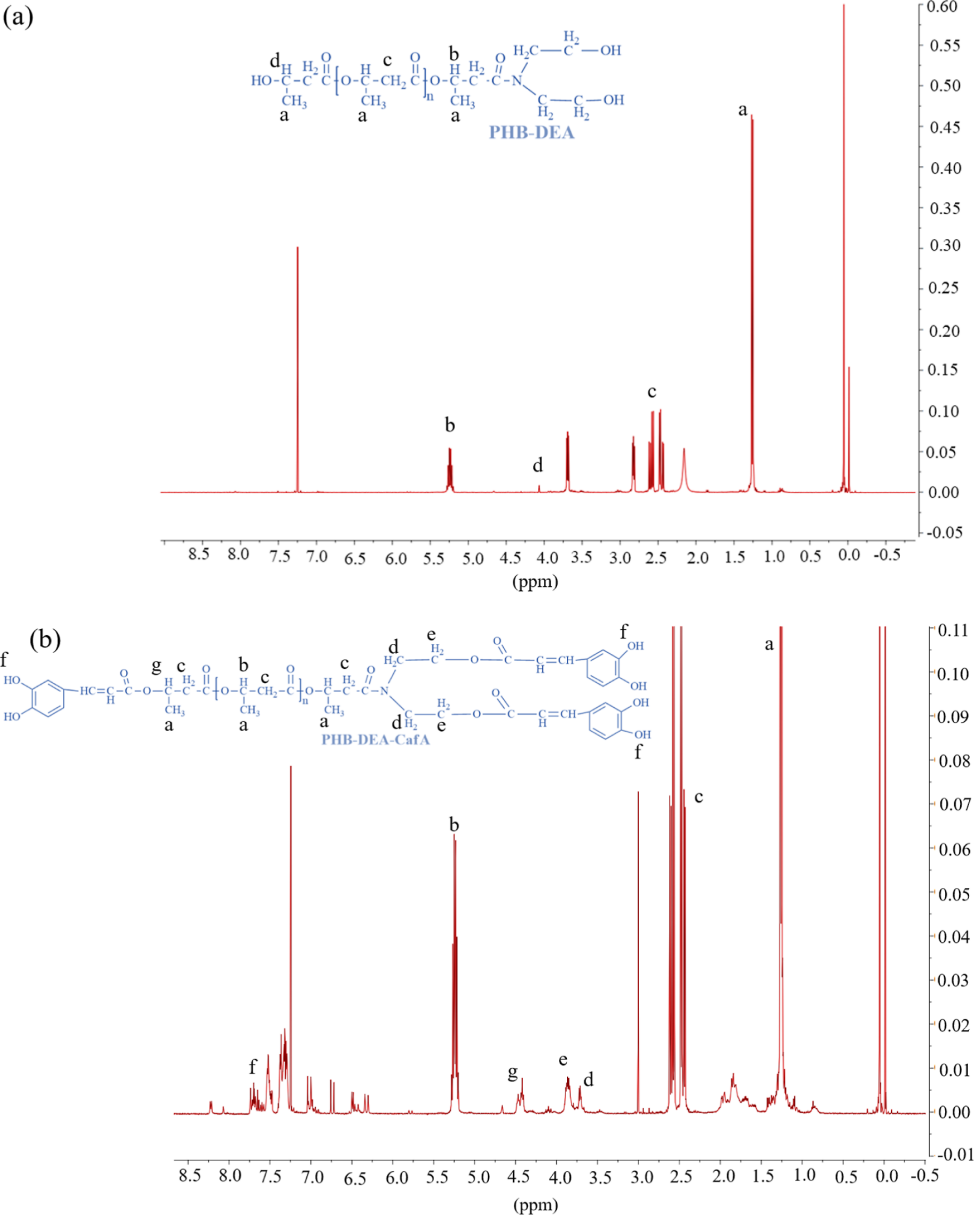
^1^H NMR spectra for (a) PHB-DEA and (b) the chain-end modified PHB-DEA-CafA.

PHB is a microbially produced polyester having one carboxylic acid and one hydroxyl end. To generate hydroxylated PHB with three hydroxyl endings, DEA was capped with the carboxylic end of PHB. Tri-hydroxylated PHB (PHB-DEA) was generated many times as a precursor in previous investigations.^25,36^ The FTIR and ^1^H NMR techniques were used to structurally characterize the resulting PHB-DEA. Characteristic PHB-DEA FTIR signals (Figure 3) were found at 1563, 3315, and 1736 cm^–1^, which corresponded to amide carbonyl, DEA primary hydroxyl groups, and PHB ester carbonyl, respectively. On another avenue, the ^1^H NMR spectra (Figure 4a) of PHB-DEA displayed the typical chemical shifts at 1.3 ppm for −CH_3_, 2.4–2.6 ppm for −CH_2_–COO–, 3.0 ppm for −N–CH_2_–, 3.5–3.8 ppm for −CH_2_–OH, 4.1 ppm for −CH–OH, and 5.1–5.3 ppm for −CH–O–.^37^

Despite the positive features of PHAs in numerous applications, they are inactive polymers. To illustrate, they do not possess biological activities due to the lack of essential functional groups in their structure.^38^ For this reason, the three hydroxyl groups of PHB-DEA were substituted with CafA molecules to enhance the polymer functionalities. Alkene and aliphatic carboxylic acid groups are the two main functional groups of caffeic acid as a cinnamic acid derivative. The FTIR spectra of CafA displayed two broad overlapping bands for O–H and C–H extending vibrations from 2400 to 3300 cm^–1^. The stretching vibrations of the carbonyl group (C=O) caused the peak of the carboxylic acid group at 1685 cm^–1^. This C=O absorption band was significantly distinguished from the alkene C=C stretching absorption band at 1634 cm^–1^. The benzene ring’s C=C stretching vibrations had two peaks at 1590 and 1514 cm^–1^.^39^ The characteristic absorbances for these alkene and aliphatic carboxylic acid groups remained apparent in the spectra of the synthesized PHB-DEA-CafA at 1630 and 1688 cm^–1^ (Figure 3). Moreover, at 1585 and 1500 cm^–1^, the arene C=C stretching vibrations of the benzene ring were observed (Figure 3). The structure of PHB-DEA-CafA was also confirmed by the ^1^H NMR (Figure 4b), where the chemical shifts at 1.3 ppm for −CH_3_, 2.4–2.6 ppm for CH_2_–COO–, 3.0 ppm for −N–CH_2_–, 3.5–3.8 ppm for −CH_2_–OH, 4.1 ppm for −CH–OH, 5.1–5.3 ppm for −CH–O–, 7.086 ppm for −C=H, and 7.4–7.55 ppm for cyclic–C–H were observed. The hydroxyl groups of PHB were previously substituted by *N*-isopropyl acryl amide (NIPAM), forming a thermoresponsive block copolymer for biomedical applications.^25^ Similarly, Erol et al. studied PHB-DEA reaction with 2,3,5-tri-iodobenzoic acid and 4-iodobenzoic acid, generating tri-novel radiopaque iodinated PHB with X-ray visibility for medical diagnosis.^36^ The previously mentioned studies were proposed with the aim of enhancing the biomedical functionality of PHB. However, the current work investigates the potentials of PHB functionalized with natural molecules (CafA) for the food sector; especially active food coatings. Regarding the molecular weight analysis, the GPC results showed that the extracted PHB had a molecular weight of 187,000 Da (PDI 2.5), while this value dropped to 4700 Da (PDI 1.5) for PHB-DEA after transamination. Furthermore, the newly synthesized PHB-DEA-CafA had a molecular weight of 6900 Da (PDI 1.75). Therefore, it was concluded that the data obtained based on FTIR, ^1^H NMR, and GPC analysis affirmed a successful synthesis of PHB-DEA-CafA, which was next examined for its thermal stability compared to the native polymer.

### Thermal Analysis

**Figure 5:**
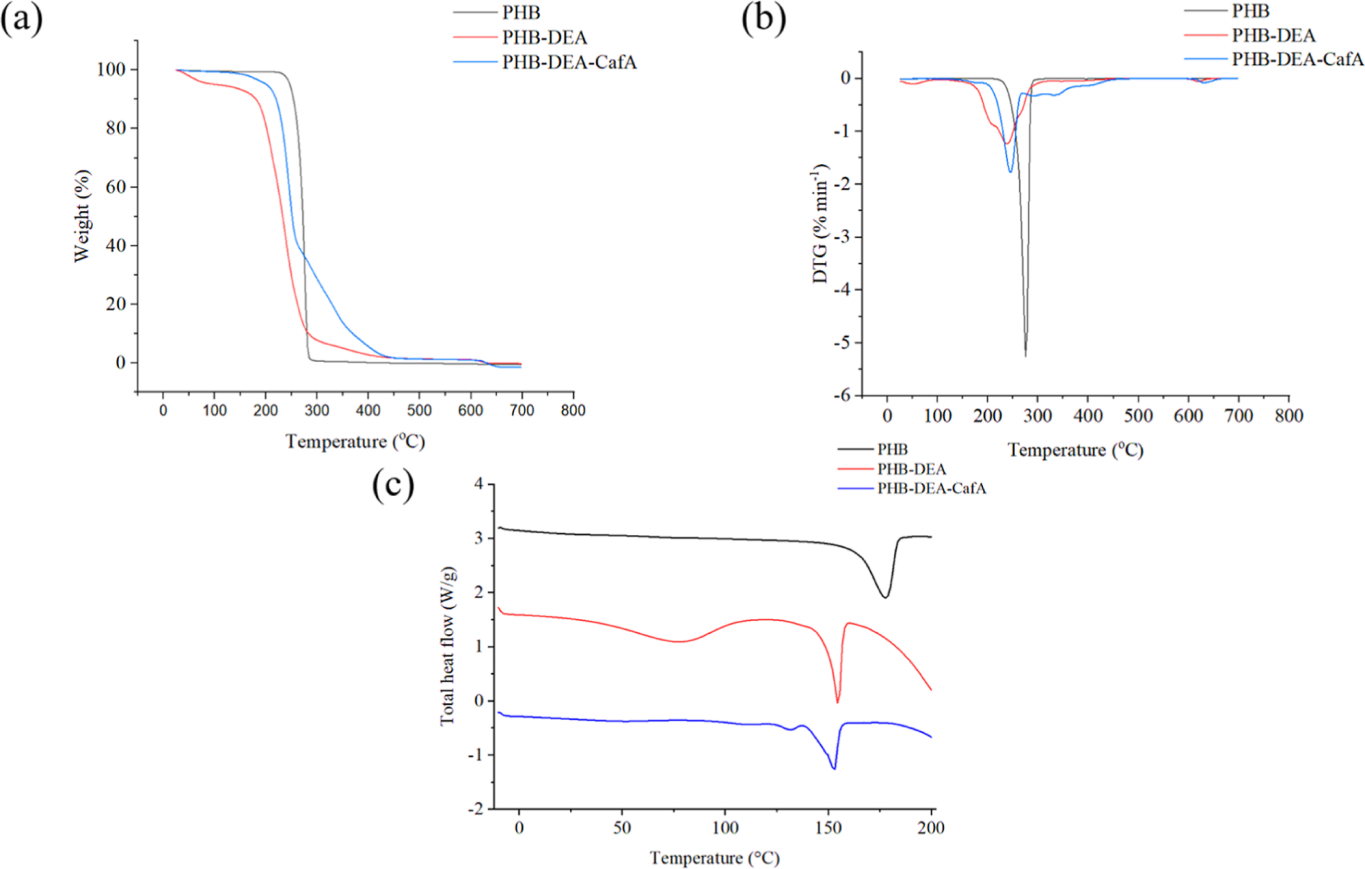
Thermal analysis of PHB, PHB-DEA, and PHB-DEA-CafA including (a) TGA, (b) DTG, and (c) DSC.

The TGA profiles of PHB, PHB-DEA, and PHB-DEA-CafA were depicted in Figure 5a. The TGA curves indicated the weight loss occurred in two stages for the three polyester types. The first step of mass loss for all polymer samples was detected at temperatures ranging from 90 to 180 °C. This mass loss was roughly 1.5% of PHB mass, 5% for PHB-DEA, and 2.4% for PHB-DEA-CafA, due to evaporation of adsorbed solvents. The second significant step in the degradation of all polymers occurred after 200 °C, that was above the standard PHB melting point.^40^ In fact, the degradation process includes a drop in molecular weight due to hydrolysis and chain scission of the polymeric backbone. The random chain scission process is responsible for the rapid thermal breakdown of polyesters at this stage, which involves the cleavage of C–O and C=O bonds and the destruction of crystalline regions.^41^ It was obviously reported that the maximum degradation temperature for PHB, PHB-DEA, and PHB-DEA-CafA were 290, 262, and 285 °C, respectively. Thus, it is clear that PHB-DEA-CafA had almost the same thermal stability as native PHB, while PHB-DEA was the least thermally stable polymer. It should be noted that the residual mass of PHB, PHB-DEA, and PHB-DEA-CafA, respectively, was less than 3%. It is noteworthy that, functionalization of PHB had no negative impact on its thermal properties.

DTG analysis was used to assess the rate of mass loss of a polymer sample in proportion to temperature. Figure 5b shows the DTG analysis for PHB, PHB-DEA, and PHB-DEA-CafA. The peaks in the DTG curves reflect the thermal stability of various polyesters with respect to the fastest polymer degradation temperature. The mass loss rates for PHB were roughly 0.12 to 0.25%/min in the first phase, 0.49 to 0.88%/min for PHB-DEA and 0.19 to 0.37%/min for PHB-DEA-CafA up to 190 °C. Furthermore, the PHB’s highest degradation temperature was at 290 °C, with a maximum mass loss rate of 35%/min in the second degradation phase, while the maximum degradation temperature for PHB-DEA was around 262 °C with a maximum mass loss rate of 46%/min. Hence, PHB-DEA demonstrated less thermal resistance to degradation as compared to native PHB. For PHB-DEA-CafA, the highest degradation temperature was observed to be around 285 °C, with the highest value of 40%/min for mass loss rate. PHB-DEA-CafA had a higher thermal resistance to deterioration and a slower mass loss rate compared to PHB-DEA.^40^

In addition to this, to demonstrate the impact of the polymeric functionalization on the thermal behavior of the polymer, DSC of PHB, PHB-DEA, and PHB-DEA-CafA are shown in Figure 5c. The glass transition (*T*_g_) and the first (*T*_m1_) and second melting (*T*_m2_) temperatures of PHB were −5.1, 150.3, and 165.2 °C, respectively. While for PHB-DEA, the *T*_g_, *T*_m1,_ and *T*_m2_ were −8.1, 139.2, and 151.3 °C, respectively. The *T*_g_, *T*_m1,_ and *T*_m2_ were −5.7, 145.7, and 162.8 °C, respectively, for PHB-DEA-CafA. The molecular weight of polymers was previously reported to be positively correlated with its *T*_g_ value.^42^ Single *T*_g_ value for PHB-DEA and PHB-DEA-CafA, respectively, in the amorphous phase indicates an efficient reaction between the two main components.^43^ Furthermore, the presence of CafA had little effect on *T*_g_, which is less than 1 °C. The presence of two crystalline phases in PHB-DEA-CafA was shown by the two endothermic peaks displayed in Figure 5c. The incorporation of CafA reduced the two melting temperatures compared to PHB and PHB-DEA, demonstrating that PHB-DEA and CafA have efficiently interacted forming PHB-DEA-CafA.^43^ For all samples, primary and secondary crystallization processes produced two endothermic peaks in the curves. These bimodal melting peaks provided additional evidence for the presence of melt-recrystallization processes.^44^

### Biodegradation Studies

**Figure 6:**
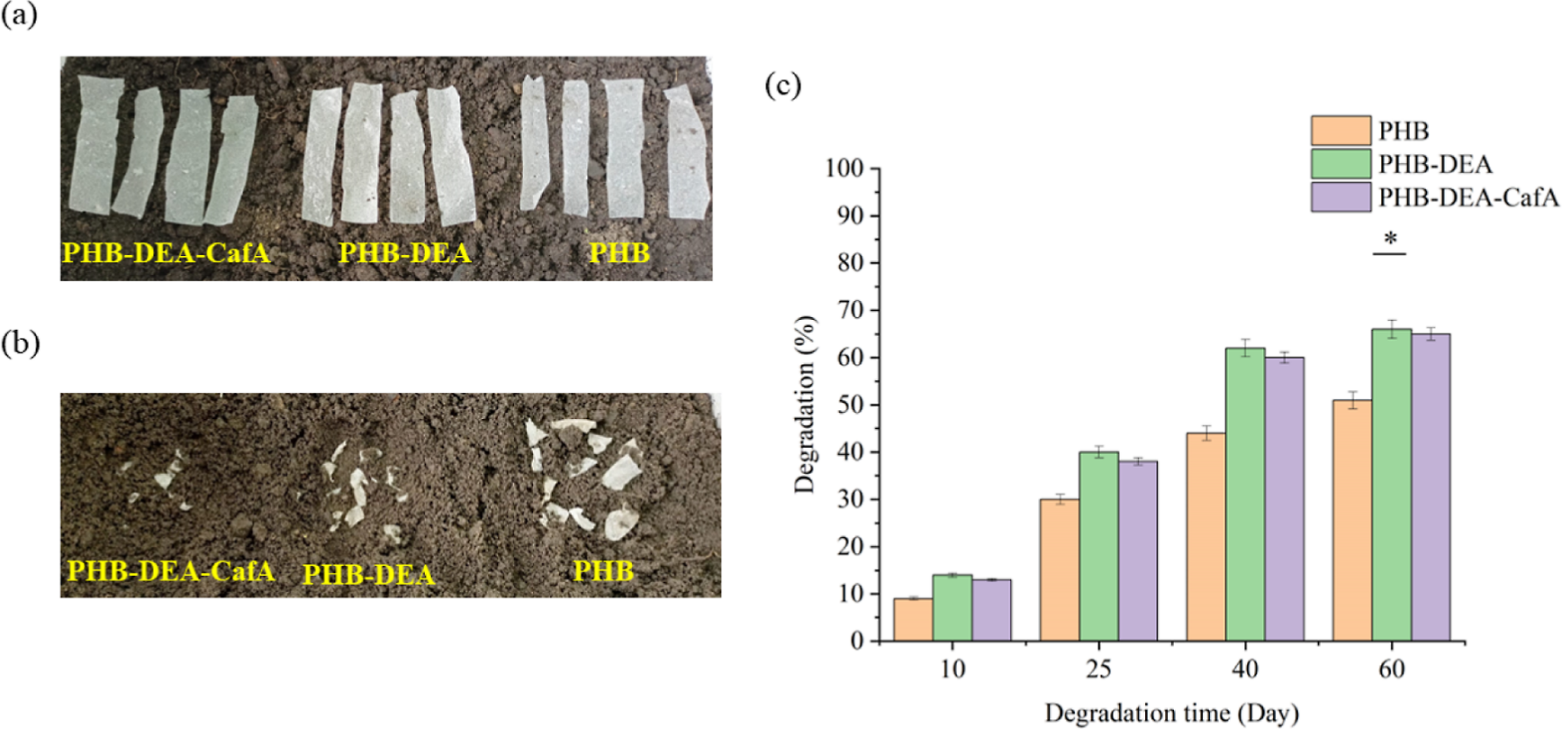
Representative images of PHB, PHB-DEA, and PHB-DEA-CafA films illustrating (a,b) the biodegradation profile after 60 days as well as (c) the degradation percentage over 60 days in clay soil at 25 °C.

In particular, compared to other biopolymers, PHB has the benefit of degrading in both aerobic and anaerobic environments,^45^ resulting in a rapid decomposition rate and consequently a lower environmental impact. The temperature of the microenvironment was set at 25 °C during the biodegradation study. This temperature was close to the ideal range for mesophilic bacteria (25–40 °C) and fungi (22–30 °C) responsible for the biodegradation process.^46^ The biodegradation of three polymer strips was monitored within 60 days. From day 40, the degradation rates were higher than 40% for the entire polymeric strips. The degradation rate of PHB films was slower than those of the other polymeric matrices (Figure 6a,b), with a maximum degradation percentage of 51% on day 60. Similarly, the degradation percentages of PHB-DEA and PHB-DEA-CafA strips were 66 and 65%, respectively, on day 60 (Figure 6c). The slight decrease in the percentage of biodegradation of PHB-DEA-CafA could be attributed to its high molecular weight compared to PHB-DEA.^47^ Also, it could be related to the antimicrobial efficacy of the CafA constituents, which could affected the soil microbial community.^48^

### Physicochemical Characterization of PHB-DEA-CafA NPs

**Figure 7:**
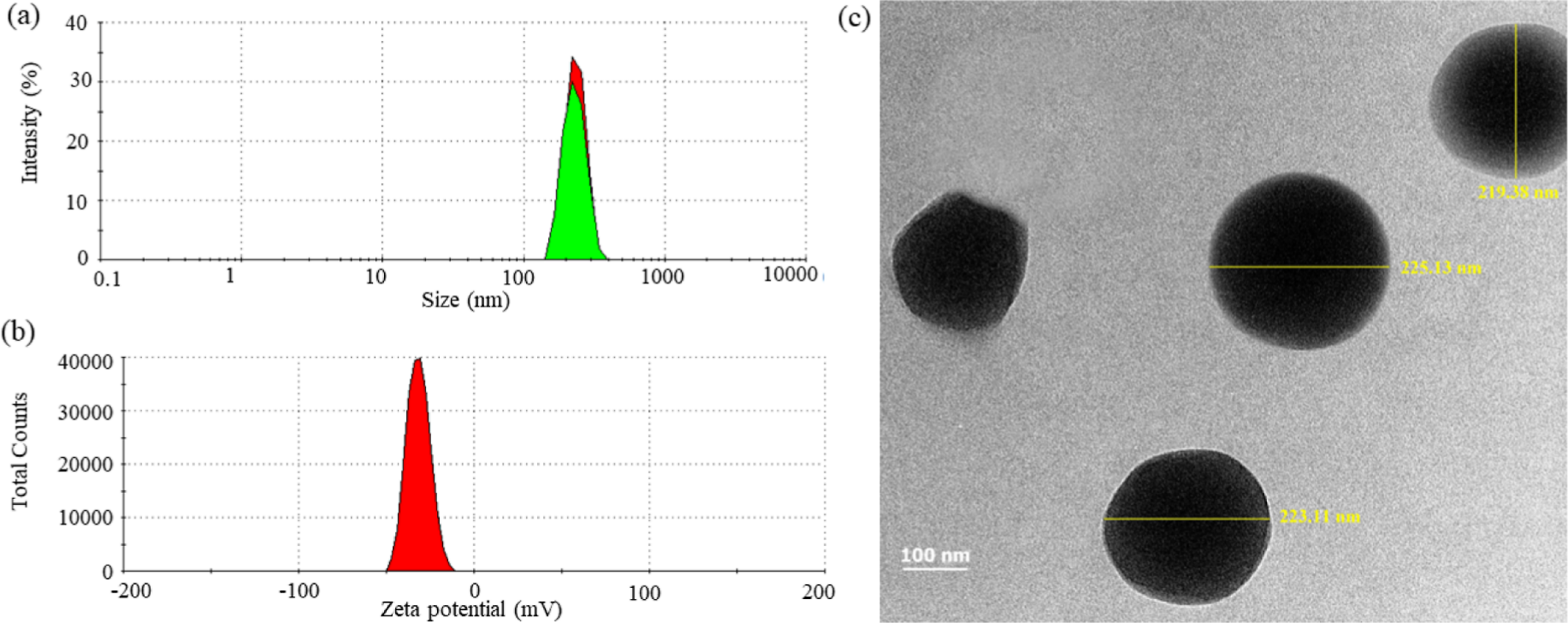
Physical characterization of PHB-DEA-CafA NPs including (a) particles size distribution, (b) zeta potential, and (c) morphology determination by TEM.

CafA encapsulation or integration in functional platforms has lately gained popularity due to its prospective uses in the food industry.^49,50^ Several processes are used to create polymeric NPs, including emulsion-solvent evaporation,^51^ electrostatic interaction,^52^ and nanoprecipitation.^53^ Generally, the synthesis of PHAs NPs requires the dissolution of the polyester in an organic solvent such as dichloromethane, chloroform, acetone, etc. However, many industries have moved toward employing more eco-friendly or green solvents in recent years when it comes to biopolymer extraction and chemical recycling.^54^ In the present work, acetic acid was used as an alternative solvent for PHB-DEA-CafA to produce the green NPs. Transforming the newly synthesized polymeric matrix to the nanoscale sizes increases the surface-area-to-volume ratio significantly, which accelerates dissolution and diffusion rates.^55^ We were able to obtain PHB-DEA-CafA NPs with mean particle sizes of 223 ± 0.12 nm, zeta potential of −31 ± 0.25 mV, and PDI of 0.25 ± 0.03 (Figure 7a,b). PHB-DEA-CafA was reduced to the nanoscale aiming at the antibacterial and antioxidant efficiency of the functionalized polymer. This correlated to the fact that NPs have the ability to penetrate and accumulate within bacterial cells. Furthermore, NPs were shown to be powerful tools for penetrating the bacterial cell wall peptidoglycan layer, creating pores, and inducing death.^56^ The size of the NPs generated in this study was appropriate for demonstrating significant antibacterial properties. When it came to particle charge and PDI, the NPs had a surface charge of −31 ± 0.25 mV and PDI of 0.25 ± 0.03. The negative charge is likely related to the hydroxyl groups in the polymeric backbone, and the high value of zeta potential indicates strong colloidal stability.^26^ The size of PHB-DEA-CafA NPs was in the same range as that of those prepared by Corrado et al.^57^ who recently generated NPs based on PHB and poly-3-hydroxybutyrate-*co*-hydroxyhexanoate (PHB-HHx), encapsulated with Mexican oregano essential oil with mean particles size of 210 nm to exhibit a potent antibacterial activity against *Micrococcus luteus*.^57^ PHB-DEA-CafA NPs demonstrated a spherical to oval shape when investigated by TEM. The mean particles sizes recorded by Malvern Zetasizer agreed with values obtained based on TEM analysis (Figure 7c). Furthermore, there was no particle aggregation or accumulation, which explained why the zeta potential was relatively high and the PDI was low.

### In Vitro Studies

#### Antioxidant Capacity of PHB-DEA-CafA NPs

**Figure 8:**
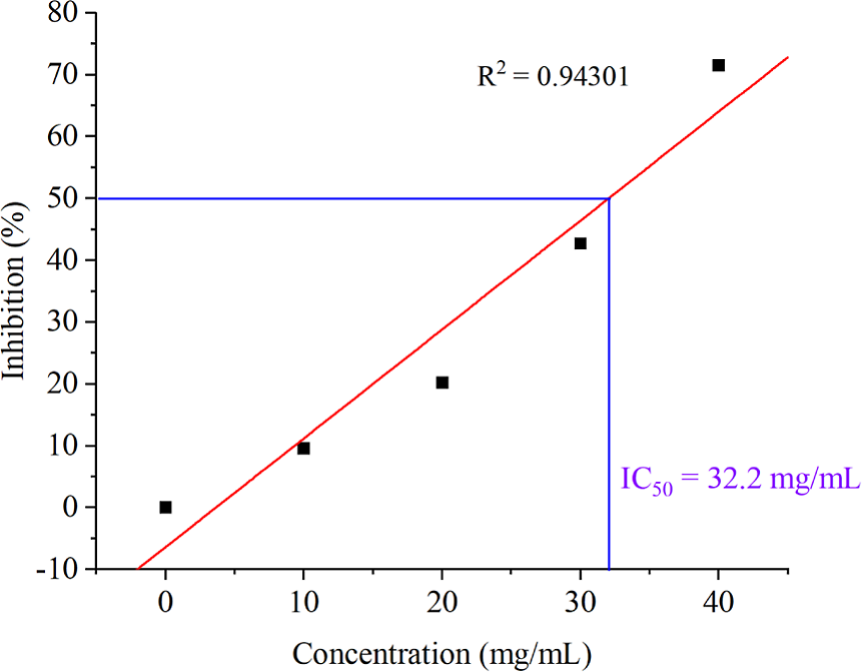
Antioxidant activity of PHB-DEA-CafA NPs using DPPH free radical scavenging activity.

**Figure 9:**
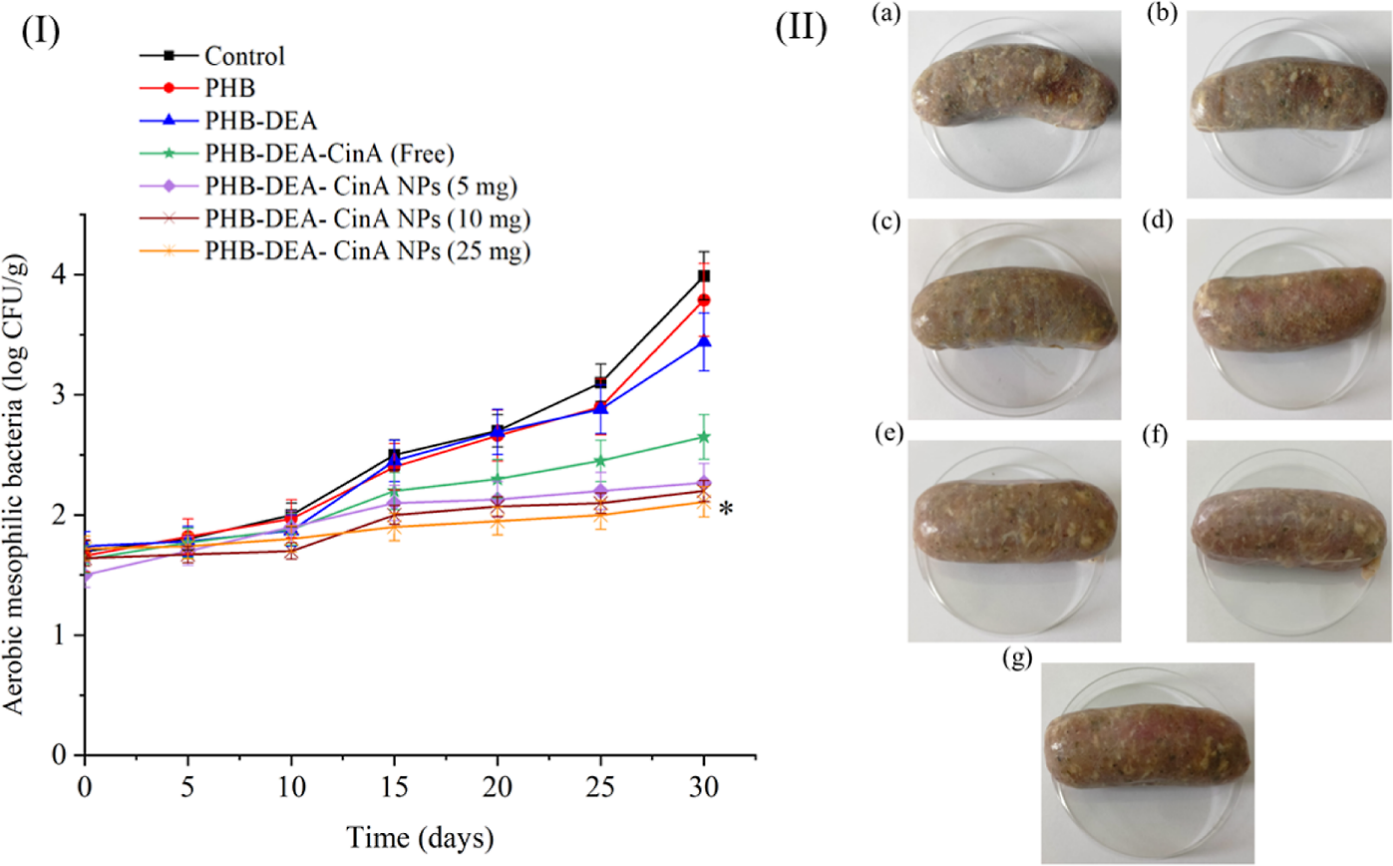
(I) Microbial analysis (TBC) of raw Polish sausages using different treatments over 30 days of storage at 4 °C as well as (II) representative images for (a) control, (b) PHB, (c) PHB-DEA, (d) free PHB-DEA-CafA (5 mg), (e) PHB-DEA-CafA NPs (5 mg), (f) PHB-DEA-CafA NPs (10 mg), and (g) PHB-DEA-CafA NPs (25 mg) after 30 days of storage at 4 °C.

Antioxidants, which can be natural or synthetic, are additives that protect food from oxidative degradation during storage and processing. Because of their low volatility and high stability, antioxidants contribute in the preservation of freshness, color, taste, functionality, nutrients, texture, and consumer appeal.^58^ Due to the advantageous features of phenolic acids, they have been attached to numerous polymeric structures to confer additional properties on polymers, consequently increasing their applications.^59^ The antioxidant activity of phenolic acids has been used to generate antioxidant polymers. To increase its antioxidant potential, chitosan was modified with phenolic acids. Such polymers have been used as food contact surfaces to reduce fat oxidation.^60^ The CafA content in PHB-DEA-CafA chains was determined to be 50 mg/mL, which reflects the high substitution efficiency in the chemical modification reaction between PHB-DEA and CafA molecules. The DPPH assays of PHB-DEA-CafA NP solutions are displayed in Figure 8. The percentage inhibition was found to be concentration dependant. PHB-DEA-CafA NPs with a concentration of 10 mg/mL exhibited low 9.6% inhibition, while 40 mg/mL of the respective NPs could inhibit incredibly 71.8% of DPPH. The sample concentration that resulted in a 50% reduction in total DPPH radicals (IC_50_) was reported to be 32.2 mg/mL. Such high antioxidant capacity of the modified PHB NPs would be of great significance when it comes to food preservation applications.^61^ The antioxidant capacity of the investigated NPs could be attributed to the presence of CafA molecules in the functionalized developed polymer. The high antioxidant capacity of CafA may be due to the presence of phenolic hydroxyl groups in the benzene ring, which are required for significant superoxide anion scavenging.^62,63^ Packaging films based on ethylene vinyl alcohol copolymer (EVOH) and CafA were obtained by Luzi et al.^64^ According to the authors, the inclusion of active CafA in the polymeric films ameliorated the free radical scavenging performance, demonstrating the feasibility of using these polymeric systems in the food packaging sector. Our findings were also supported by Zeren et al.,^65^ where CafA was efficiently encapsulated in whey protein-based and carob bean flour nanofibers. CafA significantly increased the antioxidant capacity of the fabricated electrospuns to 92.95 ± 1.19%. CafA’s antioxidative action is known to be enhanced by the addition of a second hydroxyl group in the ortho- or para-position due to increased resonance stability and *o*-quinone or *p*-quinone formation.^65^ The promising antioxidant reports qualify PHB-DEA-CafA for food preservation potentials (Figure 9).

#### Antibacterial Efficacy against Food Pathogens

The antibacterial effectiveness of PHB-DEA-CafA NPs was determined using the plate counting method at various time intervals (8, 12, 24, and 48 h) compared to the positive control group. It was apparent that inhibition of bacterial cell viability was dependent on PHB-DEA-CafA NPs concentration. It was observed that after 12 h the concentration of 5 mg/mL of the NPs could significantly reduce growth (*P* < 0.05) 41 ± 0.23 and 45 ± 0.4% of *S. aureus* DSM 683 and *L. monocytogenes* DSM 19094, respectively, compared to the positive control group (Table 1). However, lower growth inhibition percentages for *S. enterica* DSM 9386 and *E. coli* DSM 787 were recorded to be 36 ± 0.3 and 32 ± 0.1%, respectively, at the same concentration and time interval. In particular, after 48 h of exposure, active NPs resulted in a remarkable growth inhibition percentage of 98 ± 0.12% for *L. monocytogenes* DSM 19094, while the least sensitive strain was *E. coli* DSM 787 with a growth inhibition percentage of 79 ± 0.23% at the end of the experiment. Gram-negative bacteria were often more resistant to the generated PHB-DEA-CafA NPs than Gram-positive bacteria, which is likely related to the structure of Gram-negative bacteria, which is highly selective for hydrophilic compounds.^52^ The inverse relationship between incubation duration and cell viability percentage is ascribed to prolonged exposure of bacterial cells to functional NPs. The potent antibacterial capacity of the investigated NPs against Gram-positive and Gram-negative food pathogens could be explained based on the incorporation of antibacterial CafA molecules to the trihydroxylated PHB chains.^66^ CafA has also been studied as a food preservative against food pathogens, where the existence of multiple hydroxyl groups on the benzene ring is ascribed to the antibacterial action.^11,67^ Some of the possible antibacterial modes of action of CafA could be enhancing the cell membrane permeability, damaging cell membrane integrity, reducing efflux activity, disrupting the active transport system as well as cell integrity, and inhibiting the bacterial RNA polymerase enzyme.^68−70^ Our results are in line with Wang et al., who enhanced the efficiency of chitosan films by incorporating CafA in its chains through chemical grafting. The grafted CafA-chitosan films had the capability of inhibiting 92.22 ± 2.54 and 88.07 ± 0.93% of *S. aureus* and *E. coli*, respectively.^49^ The authors also recommended the grafted films for active food packaging applications. Remarkably, the presented PHB-DEA-CafA NPs could inhibit at least 80% of all bacterial cultures over a period of 48 h.

**Table 1:** Antibacterial Efficiency of PHB-DEA-CafA NPs against Different Bacterial Strains^a^

bacterial strains												
percentage of growth inhibition (%) after exposure to different concentrations of PHB-DEA-CafA NPs (mg/mL)												
	S. aureus DSM 683			L. monocytogenes DSM 19094			S. enterica DSM 9386			E. coli DSM 787		
time (h)	1	2.5	5	1	2.5	5	1	2.5	5	1	2.5	5
8	4 ± 0.13^aA^	11 ± 0.4^aB^	20 ± 0.1^aC^	5 ± 0.22^aA^	13 ± 0.11^aB^	23 ± 0.35^aC^	3 ± 0.11^aA^	6 ± 0.25^aA^	15 ± 0.15^aB^	3 ± 0.15^aA^	6 ± 0.22^aA^	13 ± 0.05^aB^
12	7 ± 0.22^aA^	20 ± 0.15^bB^	41 ± 0.23^bC^	9 ± 0.14^aA^	25 ± 0.16^bB^	45 ± 0.4^bC^	8 ± 0.22^aA^	18 ± 0.23^bB^	36 ± 0.3^bC^	7 ± 0.19^aA^	17 ± 0.2^bB^	32 ± 0.1^bC^
24	20 ± 0.3^bA^	45 ± 0.2^cB^	72 ± 0.11^cC^	17 ± 0.26^bA^	39 ± 0.24^cB^	77 ± 0.15^cC^	14 ± 0.03^bA^	30 ± 0.14^cB^	69 ± 0.12^cC^	13 ± 0.06^bA^	27 ± 0.25^cB^	61 ± 0.25^cC^
48	28 ± 0.1^cA^	52 ± 0.18^dB^	95 ± 0.05^dC^	26 ± 0.23^cA^	58 ± 0.05^dB^	98 ± 0.12^dC^	24 ± 0.33^cA^	36 ± 0.11^dB^	83 ± 0.2^dC^	19 ± 0.1^cA^	29 ± 0.6^dB^	79 ± 0.23^dC^

a After incubating the cultures at 37 °C for 18–24 h, aliquots were dispersed on TSA plates to determine the bacterial count. Three independent experiments provided data. Small superscripts with different letters in the same row mean significance (*P* < 0.05), while capital superscripts with different letters in the same column mean significance (*P* < 0.05).

#### Microbiological Analysis of Raw Commercial Polish Sausage

Because the generated NPs demonstrated promising antioxidant and antibacterial efficacies in vitro, they were examined for active food coating applications. The raw sausage samples were coated with six different coatings including PHB, PHB-DEA, free PHB-DEA-CafA, and PHB-DEA-CafA NPs with three different concentrations, while the control group received no treatment. On day 0, the TBC for all samples ranged from 1.5 ± 0.01 to 1.74 ± 0.05 log cfu/g. The control group witnessed a significant increase (*P* < 0.05) in the bacterial count of 2.0 ± 0.11 and 2.5 ± 0.03 log cfu/g after 10 and 15 days of storage at 4 °C, respectively. On the other hand, samples coated with PHB-DEA-CafA NPs with the highest concentration of 25 mg/mL had low TBC of 1.9 ± 0.02 log cfu/g on day 15. PHB and PHB-DEA coatings exhibited almost no antibacterial activity or a considerable effect on TBC as the records were comparable to those of the positive control group. However, the most active coatings were free PHB-DEA-CafA and three concentrations of PHB-DEA-CafA NPs. It should be emphasized that PHB-DEA-CafA NPs displayed superior antibacterial effect when compared to the free counterpart at the same concentration. The reason behind this could be related to the high surface-area-to-volume ratio of the NPs, which triggers more capability of penetrating the peptidoglycan-based bacterial cell wall.^52^ Transforming the polymeric matrix to the nanoscale exposes the bacterial cells to more functional groups, thereby enhancing their efficacy.^52^ The lowest bacterial count and the most satisfactory coating was 25 mg/mL PHB-DEA-CafA NPs with 2.11 ± 0.01 log cfu/g compared to 3.99 ± 0.13 log cfu/g of the positive control group on day 30. From the obtained results, it is apparent that 25 mg/mL PHB-DEA-CafA NPs coatings could hinder the microbial growth over 30 days compared to the other coatings, which results in prolonging the shelf life of such commercial products in the market. Nevertheless, sausage samples treated with other coatings spoiled much faster over the same time period, which reflects poor samples safety for customer consumption.

Our investigated coatings were much more effective than the active films proposed by Qiu and Chin, who wrapped raw pork sausages with a package of sodium alginate based on lotus rhizome root powder. A TBC of 3.9 ± 0.48 log cfu/g was recorded for the control group against 3.39 ± 0.31 log cfu/g for sodium alginate films incorporated with 1% oven-dried lotus rhizome root powder after 35 days of storage.^71^ Recently, CafA has also been grafted to the backbone of chitosan and formulated as an active coating for Pompano fish preservation. The synthesized coatings showed effective antioxidant and antibacterial capacities, maintaining the quality of the Pompano flesh.^72^ When the previous data are considered, PHB-DEA-CafA NPs are highly recommended for controlling the microbial burden in sausage products over longer periods compared to conventional polymers.

## Conclusions

The new local PHB producer *B. nealsonii* ICRI16 has been isolated with 97% similarity to *B. Nealsonii* species. CafA reacted effectively with the hydroxylated PHB form in the presence of DCC catalyst and Argon atmosphere environment, generating novel PHB-DEA-CafA, whose structure was confirmed by FTIR and NMR. The developed polymer had relatively lower molecular weight compared to the native PHB. Importantly, the functionalization process did not decrease the thermal tolerance of the newly synthesized polyester in comparison with PHB. In addition, nanoparticulate modified polyester was generated using a green approach by dissolving PHB-DEA-CafA in acetic acid to avoid hazardous reducing agents for greater suitability in the food industry. These respective NPs exhibited effective in vitro antioxidant efficacy to retard lipid oxidation and enhance food quality. Additionally, more than 60% of Gram-positive and Gram-negative bacterial populations were inhibited in vitro after 24 h of exposure. It should be noted that polymeric-based NPs had a highly positive effect on the microbial quality of meat/pork sausage over 30 days of storage. Therefore, such materials could be considered as good candidates for functional foods and food industry applications.

## Future Prospects

The current study is highly recommended for wider industrial implementation as commercial active food coatings with no additional active ingredients since the polymer itself demonstrates required functionality. The co-friendliness of the generated bioplastic-based coating would be another factor for boosting green environment strategies. Furthermore, the obtained results open the door to investigate the use of the proposed coatings on other food samples such as sea food, fruits, and different types of meats in terms of assessing the microbial load and food quality. Finally, PHB-DEA hydroxyl groups’ substitution with other active phenolic acids or molecules could be investigated for ameliorating the polyester bioactivities and hence possessing versatile potentials.

## Notes

The accession number(s) can be found on the following GenBank site: https://www.ncbi.nlm.nih.gov/, ON231791.
